# Quantifying Inequalities in Childhood Immunization Using Summary Measures of Health Inequality: An Application of WHO Stata and R ‘Healthequal’ Packages

**DOI:** 10.3390/vaccines12121324

**Published:** 2024-11-26

**Authors:** Katherine Kirkby, Daniel A. Antiporta, Anne Schlotheuber, Patricia Menéndez, M. Carolina Danovaro-Holliday, Ahmad Reza Hosseinpoor

**Affiliations:** 1 Department of Data and Analytics, World Health Organization, 20 Avenue Appia, 1211 Geneva, Switzerland; 2 School of Mathematics and Statistics, University of Melbourne, Parkville, Melbourne, VIC 3010, Australia; 3 Department of Immunization, Vaccines, and Biologicals, World Health Organization, 20 Avenue Appia, 1211 Geneva, Switzerland

**Keywords:** health inequality, health equity, health disparity, Stata, R, summary measures of inequality, disaggregated data, monitoring, immunization, computational methods

## Abstract

**Background:** Monitoring immunization inequalities is crucial for achieving equity in vaccine coverage. Summary measures of health inequality provide a single numerical expression of immunization inequality. However, the impact of different summary measures on conclusions about immunization inequalities has not been thoroughly studied. **Methods**: We used disaggregated data from household surveys conducted in 92 low- and middle-income countries between 2013 and 2022. Inequality was assessed for two indicators of childhood immunization coverage [three doses of combined diphtheria, tetanus, and pertussis (DTP) vaccine and non-receipt of DTP vaccine or “zero-dose”] across three dimensions of inequality (place of residence, economic status, and subnational region). We calculated 16 summary measures of health inequality and compared the results. **Results:** These measures of inequality showed more similarities than differences, but the choice of measure can affect inequality assessment. Absolute and relative measures sometimes produced differing results, showing the importance of using both types of measures when assessing immunization inequality. Outliers influenced differences and ratios, but the effect of outlier estimates was moderated through the use of complex measures, which consider all subgroups and their population sizes. The choice of appropriate complex measure depends on the audience, interpretation, and outlier sensitivity. **Conclusions:** Summary measures are useful for assessing changes in inequality over time and making comparisons across different geographical areas and vaccines, but assumptions and value judgements made when selecting summary measures of inequality should be made explicit in research.

## 1. Introduction

Monitoring inequalities in immunization can help measure progress toward achieving equity. Broadly defined, inequalities are measurable differences across population subgroups [1]. Monitoring inequalities helps to identify gaps in vaccine coverage across different population subgroups, identify whether immunization programmes are reaching all people, and support the assessment of the effectiveness of interventions [2,3]. Furthermore, immunization monitoring informs the improvement in vaccine delivery systems and addresses barriers to access. The socioeconomic conditions of a society significantly shape vaccination policy, while the lack of an effective vaccination policy further deteriorates public health and economic stability, creating a self-perpetuating cycle. Systematic monitoring of inequalities is essential to inform interventions aimed at disrupting this cycle. The Immunization Agenda 2030 outlines a global strategy to achieve equitable access to vaccines and ensure that everyone, regardless of their background or location, receives the necessary immunizations [4]. In addition to coverage, a key indicator of the IA2030 is the number of zero-dose children, defined as children who are left unvaccinated. 

Selecting appropriate measures of inequality requires an understanding of their relative advantages and disadvantages, as well as the consideration of methodological issues. Summary measures of health inequality are used to express the magnitude and direction of inequality in a single number [5]. This is useful for monitoring inequality across different geographical areas and indicators, as well as monitoring changes in inequality over time to inform the development of policies and interventions [6,7,8]. Summary measures use either disaggregated data (indicator estimates broken down by population subgroups) or individual-level (micro) data as input. There is little consensus on the ideal measure for quantifying inequalities. Difference and ratio are simple summary measures that make pairwise comparisons between two population subgroups, while complex summary measures of inequality consider all population subgroups to assess inequality.

A scoping review of multi-country studies on inequalities in child vaccine coverage conducted between 2013 and 2023 found that inequalities in immunization coverage exist by household wealth, mother’s education, ethnicity, occupation, religion, urban/rural place of residence, and subnational region [9]. Along with difference and ratio, the slope index of inequality (SII) and relative concentration index (RCI) were among the most commonly used measures to characterise inequalities. However, none of the studies identified in the scoping review investigated how the choice of summary measure can lead to different conclusions regarding inequalities in childhood immunization. Comparisons of summary measures of health inequality have been published by Wagstaff (1991), Machenbach (1997), and Harper (2005) [10,11,12]. 

Packages in the statistical software R [13] and Stata [14] were published by the World Health Organization (WHO) in 2024 to assist R and Stata users in readily calculating 21 summary measures of health inequality. The R ‘healthequal’ package is available via the Comprehensive R Archive Network (CRAN) (https://cran.r-project.org/web/packages/healthequal/index.html (accessed on 13 September 2024)). The Stata ‘healthequal’ module is available via the Statistical Software Components (SSC) Archive (https://ideas.repec.org/c/boc/bocode/s459324.html (accessed on 13 September 2024)), and there are associated resources to support their use (vignettes for the R package, and a help file and step-by-step guide for the Stata package). The summary measures included in the packages reflect those that are calculated within the WHO Health Equity Assessment Toolkit (HEAT and HEAT Plus version 6.0) software application [15]. The ‘healthequal’ packages offer increased scope and flexibility for the calculation of summary measures of health inequality across different datasets and research objectives. In this paper, we highlight the utility of these new packages by conducting empirical research on the state of inequality in immunization. We use disaggregated data from multi-indicator household surveys conducted in low- and middle-income countries, analysing inequality in two immunization indicators across three inequality dimensions. The purpose of this paper is to explore how different summary measures of health inequality can be used to quantify inequality and identify if and how the choice of measure can influence conclusions. 

## 2. Materials and Methods

### 2.1. Data Sources

Data for this study come from 92 countries with a recent Demographic and Health Survey (DHS), Multiple Indicator Cluster Survey (MICS), or DHS-like national household health survey, which collects nationally representative information from children aged 12–23 or 12–35 months in low- and middle-income countries [16,17]. Disaggregated immunization coverage estimates produced from the analysis of DHS and MICS microdata by the International Center for Equity in Health at the Federal University of Pelotas were accessed from the WHO Health Inequality Data Repository (HIDR) via a Swagger Application Programming Interface (API) [18]. Subnational estimates of immunization coverage and the human development index (HDI) were sourced from the Global Data Lab Area Database [19] via the HIDR API. HDI measures the average achievements in health, education, and standard of living, with greater HDI values indicating higher human development. Additional details regarding the estimation of the subnational HDI have been published elsewhere [20]. All analyses were based on publicly available data. DHS and MICS have received ethical approval, and secondary analysis does not require further ethical review.

### 2.2. Immunization Indicators

Inequality was assessed for two immunization indicators among one-year-old children: the third dose of combined diphtheria, tetanus, and pertussis (DTP3) vaccine and non-receipt of any DTP vaccine (zero-dose). The denominator of both indicators was the number of children aged 12–23 months at the time of the survey. 

### 2.3. Dimensions of Inequality 

Dimensions of inequality refer to demographic, socioeconomic, or geographic characteristics based on which populations can be categorized into different subgroups [1]. Dimensions of inequality can be categorised as binary (with only two subgroups), ordered (with more than two subgroups that have an inherent ranking), and non-ordered (with more than two subgroups that cannot logically be ranked). In this study, three dimensions of inequality were analysed: the place of residence (a binary dimension of urban versus rural), economic status based on wealth quintiles (an ordered dimension), and subnational region (a non-ordered dimension). Subnational regions were additionally turned into an ordered dimension by ranking regions by their HDI. Wealth quintiles are based on a household wealth index, which accounts for ownership of specific assets and having access to certain services. They are constructed using principal component analysis [21]. Information about wealth quintiles is available in the DHS and MICS datasets. Place of residence and subnational region are determined within DHS and MICS surveys using country-specific criteria. 

### 2.4. Country Selection 

We selected countries for inclusion based on data availability and survey year. We used data from the latest available survey conducted between 2013 and 2022 for the analysis. Country data were excluded from calculations if data were missing for urban or rural subgroups, any wealth quintile, or more than 15% of subnational regions. See Table 1 for the number of countries included in the analyses by indicator and dimension of inequality. 

### 2.5. Statistical Analysis

We calculated relevant absolute and relative summary measures for the three dimensions of inequality [5]. While absolute measures indicate the magnitude of inequality between population subgroups, relative measures show proportional inequality. For all three dimensions, we measured absolute and relative inequality using the simple measures difference (D) and ratio (R), comparing indicator estimates in two (extreme) population subgroups. A difference of zero and a ratio of one indicate no inequality. A difference greater than zero and a ratio greater than one indicate a higher indicator estimate in urban areas, the richest quintile, the best-performing subnational region, and the region with the highest HDI. A difference of less than zero and a ratio of less than one indicates a higher indicator estimate in rural areas, the poorest quintile, and the region with the lowest HDI.

For economic status and subnational region ordered by HDI, we calculated the absolute concentration index (ACI), relative concentration index (RCI), slope index of inequality (SII), and relative index of inequality (RII). The concentration index captures the extent to which health differs across population subgroups ranked by socioeconomic status [22]. ACI measures this in absolute terms, while RCI measures this in relative terms by dividing ACI by the national average. SII and RII measure the gap between predicted indicator values between the most advantaged and most disadvantaged, taking the situation in all other subgroups into consideration. SII measures this in absolute terms (the difference between the predicted values), and RII measures this in relative terms (the ratio between the predicted values). ACI, RCI, and SII values equal to zero and RII values equal to one indicate no inequality. ACI, RCI, and SII values greater than zero and RII greater than one indicate higher indicator values among the most advantaged; ACI, RCI, and SII values less than zero and RII less than one indicate high indicator values among the least advantaged. 

For subnational region (non-ordered), we calculated the unweighted and weighted mean differences from the mean (MDMU and MDMW), unweighted and weighted indices of disparity (IDISU and IDISW), between-group variance (BGV), between-group standard deviation (BGSD), coefficient of variation (COV) and the Theil index (TI). Larger values of all these measures indicate higher levels of inequality across subnational regions. The measures equal zero if there is no subnational inequality. MDMU and MDMW show the average difference between each subnational region’s indicator estimate and the national average, with each subnational region weighted equally for MDMU and each region weighted by its population size for MDMW. IDISW is the relative version of MDMW, dividing MDMW by the national average and multiplying the result by 100. BGV summarizes all the squared deviations of the subnational estimates from the national average and is reported as the squared unit of the indicator. BGSD is calculated as the square root of BGV and is therefore reported in the same unit as the indicator. COV is the relative version of BGSD, calculated by dividing BGSD by the national average and multiplying the result by 100. TI is calculated as the sum of products of the natural logarithm of each subgroup’s share of the indicator, each subgroup’s share of the indicator, and each subgroup’s share of the population (multiplied by 1000 for easier interpretation). TI expresses inequality as a function of shares of the immunization indicator compared to shares of the population. 

To measure the total improvement expected at a national level if there was no wealth-related or subnational inequality, we calculated the population-attributable risk (PAR) and population-attributable fraction (PAF). PAR and PAF use the most advantaged subgroup or the subgroup with the best indicator value as a baseline and quantify the improvement in the national average in absolute (PAR) or relative (PAF) terms if all population subgroups had the same level of indicator as the baseline. To assess PAR across countries, the current national average and PAR were weighted by the applicable population for the indicators (children aged one year) for each country and survey year and then averaged across all countries. 

Table 2 presents an overview of the measures calculated for each dimension of inequality.

Summary measures and their 95% confidence intervals (CIs) were calculated using the ‘healthequal’ R and Stata packages. See Supplementary File S1 for the calculation formulas. All country-level results are available in Table S1. Overall levels of inequality for each indicator, dimension, and summary measure were summarized by calculating medians and the interquartile range across all countries and by World Bank 2024 income group (low-, lower-middle, and upper-middle-income) [23]. Spearman’s Rank correlation coefficient (r_s_) was calculated to compare the correlation between the various summary measure results (since not all the relationships are linear), applying thresholds of ≥0.9 for very strong correlation, ≥0.7 for strong correlation, ≥0.4 for moderate correlation and <0.4 for weak correlation [24]. 

Analyses were conducted using R version 4.4.0 and Stata 16 software; the R and Stata codes are available at https://github.com/WHOequity/healthequal_immunization (accessed on 13 September 2024). Note that the R and Stata codes can produce slightly different estimates for SII and RII due to slightly different fitting algorithms; therefore, the estimates presented within this paper are from the Stata codes. Data visualizations were developed using Tableau version 2024.1.1.

## 3. Results

### 3.1. Place of Residence Inequality

**Figure 1:**
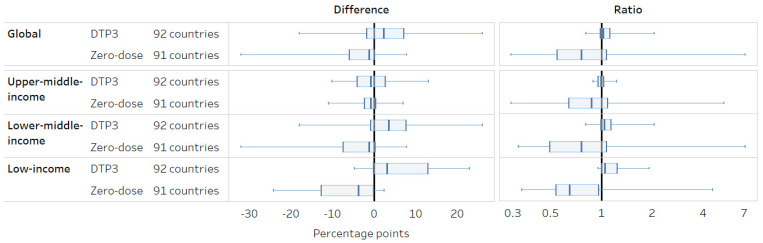
Place of residence: latest situation of inequality (2013–2022). Note: A difference of 0 and a ratio of 1 indicates no inequality. A difference > 0 and a ratio > 1 indicates a higher indicator estimate in urban areas, while a difference < 0 and ratio < 1 indicates a higher indicator estimate in rural areas.

Overall, DTP3 coverage was 2.3 percentage points (95%CI 0.4–3.6) higher in urban areas, and zero-dose prevalence was 1.2 percentage points (95%CI 0.5–2.5) lower in urban areas (Figure 1). Inequality measured using the ratio between urban and rural areas was overall low for DTP3 (1.03, 95%CI 1.01–1.04), but the urban-rural ratio in zero-dose prevalence was 0.76 (95%CI 0.65–0.87). Magnitudes of inequality differed by country income groupings for both DTP3 and zero-dose; in upper-middle-income countries, there were lower inequalities related to place of residence overall, while inequality tended to be higher and favor urban areas in most low- and lower-middle-income countries. However, there was substantial variation across countries; for instance, differences in DTP3 coverage ranged from −18 to 26 percentage points across the 92 study countries. 

**Figure 2:**
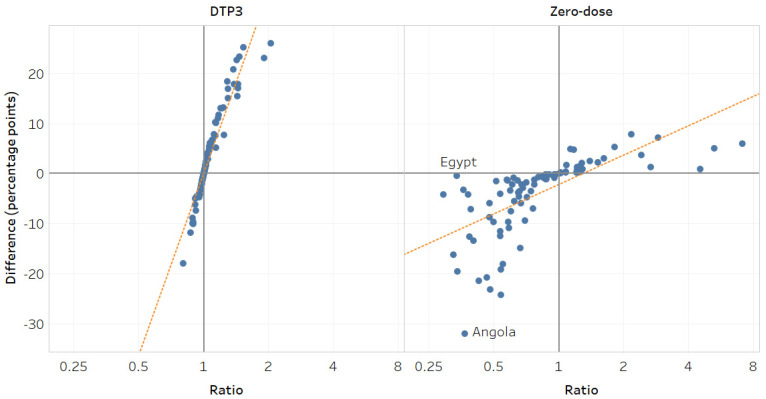
Place of residence: comparison of difference and ratio measures (2013–2022). Note: Circles represent study countries. The orange line is the line of best fit. A difference of 0 and a ratio of 1 indicates no inequality. A difference > 0 and a ratio > 1 indicate a higher indicator estimate in urban areas, while a difference < 0 and a ratio < 1 indicate a higher indicator estimate in rural areas.

There was a very strong correlation between inequality in DTP3 coverage measured using difference and ratio (r_s_ = 0.99, *p* < 0.001) and a strong correlation for zero-dose (r_s_ = 0.88, *p* < 0.001). When a country had high inequality in DTP3 coverage in absolute terms, inequality was also high in relative terms. Differences and ratios showed different magnitudes of inequality for some countries (Figure 2). For instance, urban/rural inequalities in zero-dose prevalence in Egypt (DHS 2014) and Angola (DHS 2015) were similar in relative terms, but the difference between urban and rural areas was −0.5 percentage points (95%CI −1.1–0.1) and −32.1 percentage points (95%CI −37.8–−26.4), respectively. 

### 3.2. Economic-Related Inequality

**Figure 3:**
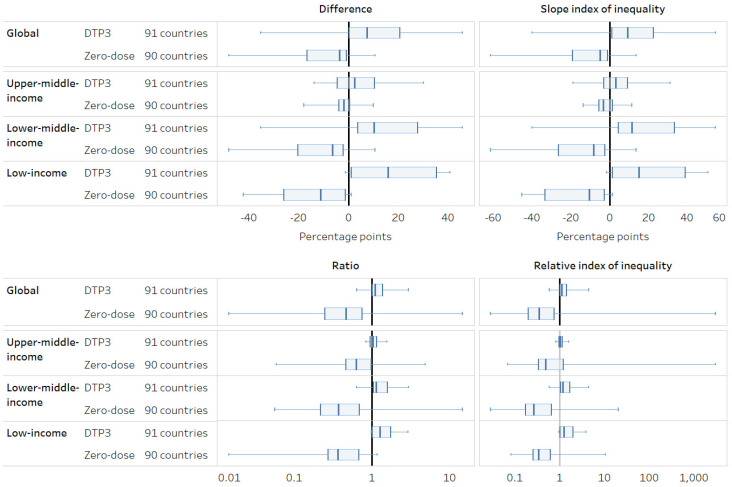
Economic status: latest situation of inequality (2013–2022). Note: A difference/SII of 0 and a ratio/RII of 1 indicate no inequality. A difference/SII > 0 and ratio/RII > 1 indicates a higher indicator estimate among the richest, while a difference/SII < 0 and ratio/RII < 1 indicates a higher indicator estimate among the poorest.

Using absolute measures of inequality (difference and SII), DTP3 coverage was overall higher among the richest quintile (D = 7.4, 95%CI: 4.0–11.0; SII = 9.2, 95%CI: 5.4–13.4) and zero-dose prevalence was overall higher among the poorest quintile (D = −3.7, 95%CI: −6.6–−2.3; SII = −4.9, 95%CI: −8.1–−3.3) (Figure 3). In relative terms, there was little inequality overall for DTP3 coverage, but zero-dose prevalence favored the richest (R = 0.47, 95%CI: 0.34–0.56; RII = 0.35, 95%CI 0.29–0.45). In upper-middle-income countries, there was little wealth-related inequality overall, both in absolute and relative terms. The patterns of inequality were similar, whether simple measures or complex measures were used to quantify the overall level of inequality. However, there was variation in the patterns and magnitudes of economic-related inequality across countries—for instance, in Tunisia (MICS 2018), DTP3 coverage was lowest, and zero-dose prevalence was highest among the richest quintile using both simple and complex measures. 

**Figure 4:**
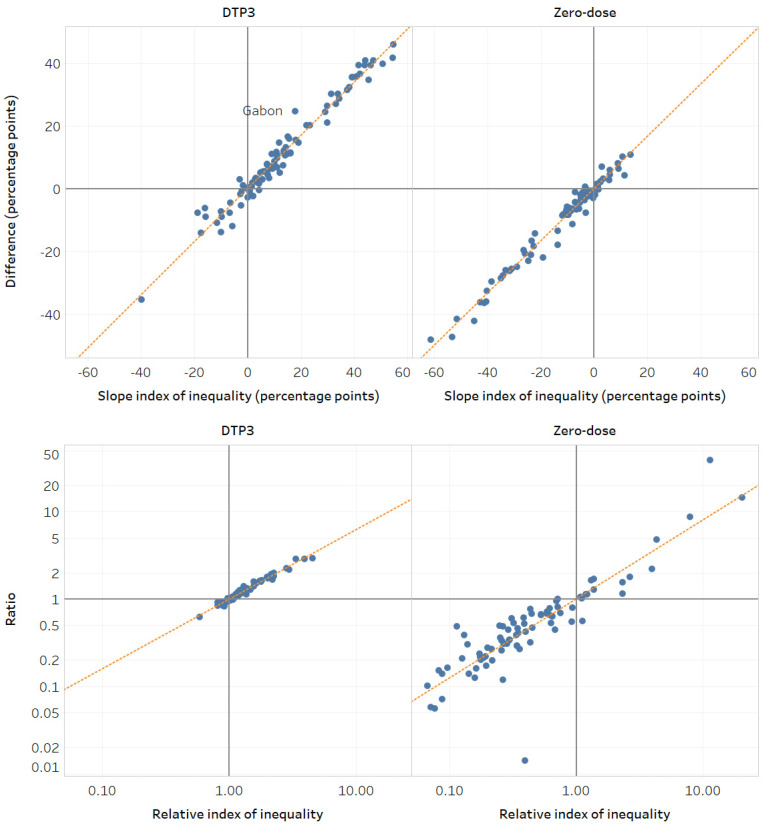
Economic status: Comparison of summary measure results across countries (2013–2022). Note: Circles represent study countries. The orange line is the line of best fit. A difference/SII of 0 and a ratio/RII of 1 indicate no inequality. A difference/SII > 0 and ratio/RII > 1 indicates a higher indicator estimate among the richest, while a difference/SII < 0 and ratio/RII < 1 indicates a higher indicator estimate among the poorest.

The level of economic-related inequality in DTP3 coverage was very strongly correlated across all the summary measures (r_s_ > 0.9, *p* < 0.001) (Table 3). However, for zero-dose prevalence, results from absolute measures were only strongly correlated with those of other absolute measures, and results from relative measures were only strongly correlated with other relative measures. SII and ACI are perfectly correlated (r_s_ > 0.99, *p* < 0.001), as are RII and RCI (r_s_ > 0.99, *p* < 0.001). Difference and ACI/SII had a strong correlation for both immunization indicators, with slight variation across countries depending on the nature of the underlying data (Figure 4). For example, DTP3 coverage in Gabon (DHS 2019) was 24.5 (95%CI 13.7–35.4) percentage points higher among the richest when measured using difference and 17.9 (95%CI −1.3–37.2) percentage points higher among the richest when measured using SII. Therefore, using SII, there was no statistically significant inequality. The discrepancy is because the difference is based on only the two extreme subgroups (quintiles 1 and 5), while SII takes all subgroups into account. 

### 3.3. Subnational Inequality

**Figure 5:**
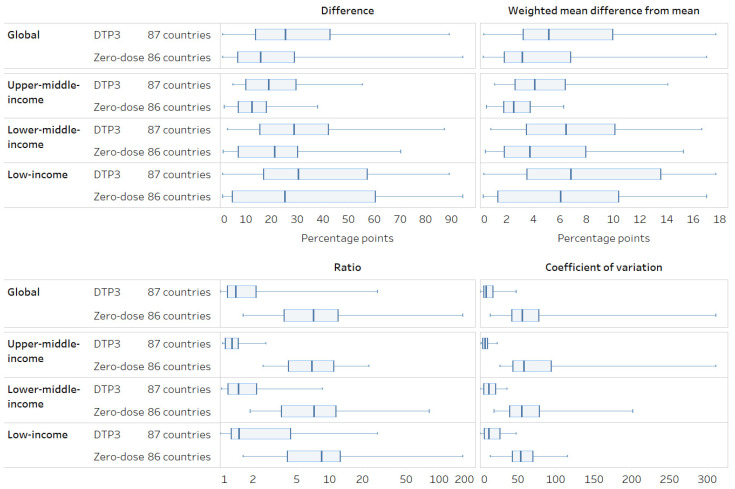
Subnational region: latest situation of inequality (2013–2022). Note: A difference/MDMW/COV value of 0 and a ratio value of 1 indicate no inequality. Higher values indicate greater inequality.

Overall, subnational inequality was measured in absolute terms using difference, and MDMW was higher for DTP3 coverage than for zero-dose (Figure 5). However, the opposite is true when measured in relative terms using ratio and COV. Absolute inequalities were highest overall in low- and lower-middle-income countries, while relative inequalities were similar across country income groups. There was substantial variation in subnational inequality across countries; for instance, the difference in DTP3 coverage between subnational regions ranged from 1 to 89 percentage points. 

While there was a strong correlation between simple measures (difference and ratio) and complex inequalities for DTP3 coverage, this was not the case for zero-dose prevalence, apart from MDMU, MDMW, BVG, and BGSD (Table 4). For DTP3 coverage, there was a strong correlation between most complex measures of inequality, while for zero-dose, there was a strong correlation only between complex absolute measures and between complex relative measures.

**Figure 6:**
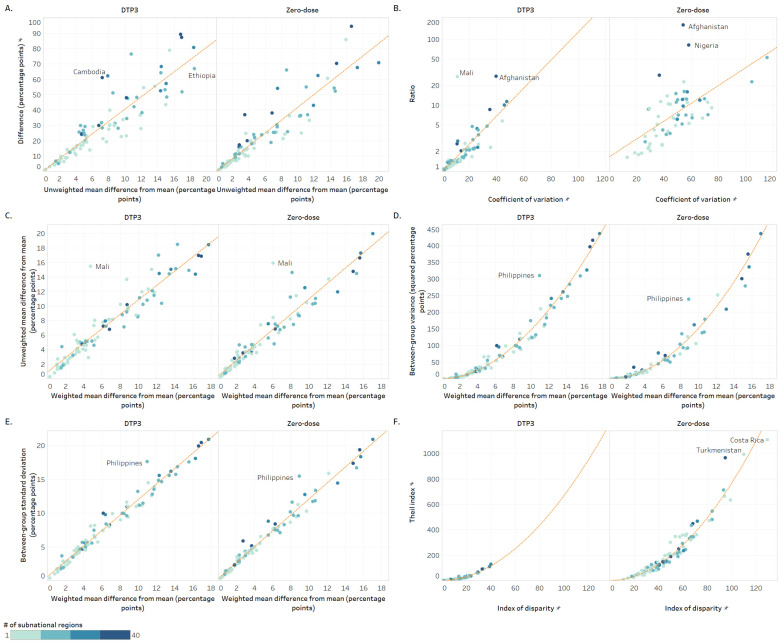
Subnational region: Comparison of summary measure results across countries (2013–2022). Note: Circles represent study countries. The orange line is the line of best fit. Higher values indicate greater inequality. (**A**) Comparison of difference against unweighted mean difference from mean; (**B**) Comparison of ratio against coefficient of variation; (**C**) Comparison of unweighted against weighted mean difference from mean; (**D**) Comparison of between-group variance against weighted mean difference from mean; (**E**) Comparison of between-group standard deviation against weighted mean difference from mean; (**F**) Comparison of Theil index against weighted index of disparity.

Although there was a strong correlation overall between difference and MDMU, at the country level, the magnitude of inequality varied (Figure 6A). For instance, Cambodia (DHS 2021) and Ethiopia (DHS 2019) had similar levels of inequality in DTP3 coverage when measured using difference (62 and 67 percentage points, respectively), but inequality measured using MDMU was higher in Ethiopia (19 compared to 8 percentage points). This is because Ethiopia had multiple regions with low DTP3 coverage, increasing the mean difference from the mean, whereas Cambodia only had one outlier region with low coverage. 

The variation between the level of inequality measured using both simple and complex measures was affected by the number of subnational regions in a country. Notably, 30 out of 49 countries (61%) with 10 or fewer subnational regions had a difference in DTP3 coverage of less than 20 percentage points, while this was the case for only 4 out of 38 countries (11%) with more than 10 subnational regions.

The ratio was more sensitive to small indicator values than COV (Figure 6B). For instance, in Afghanistan (DHS 2015), subnational zero-dose prevalence ranged from 0.7% to 82.4%, causing a ratio greater than 100. However, measuring relative inequality using COV yielded a ratio of 54, with 18 countries having higher inequality. Since the subnational region with the lowest coverage (0.7%) in Afghanistan also had a small population size, it had less influence on the weighted measure (COV). A similar situation could be observed for Mali for DTP3 coverage and for Nigeria for zero-dose. Moreover, the ratio could not be calculated when one of the two comparison subgroups had an indicator value of zero, while COV could still be calculated. 

There was a very strong correlation between MDMU and MDMW (r_s_ > 0.9, *p* < 0.001) (Figure 6C). Overall, introducing weighting by region population size decreased the magnitude of measured inequality in 69 out of 87 countries (79%) for DTP3 coverage and in 60 out of 86 countries (70%) for zero-dose. In countries where there was a small number of subnational regions and high inequality when measured using MDMU (e.g., Mali), weighting regions by population size substantially decreased the level of inequality. 

The relationship between BGV and MDMW is non-linear; rather, it can be expressed as a power regression model in which BGV is proportional to MDMW raised to a power (i.e., squared) (Figure 6D). Countries with high subnational inequality measured using MDMW had even higher subnational inequality when measured using BGV. Inequality measured using BGV had increased sensitivity to regions with extreme values; for example, The Philippines (DHS 2022) had one outlier subnational region, producing a higher BGV value. 

For both immunization indicators, there was a very strong correlation between BGSD and MDMW, both of which are weighted and measured in percentage points (r_s_ > 0.9, *p* < 0.001). There was a similarly strong correlation between the relative versions of these measures, IDISW and COV. In all countries, absolute inequality measured using MDMW was slightly higher than when using BGSD (ranging from 6.6 to 0.1 percentage points higher). Like BGV, countries with outlier regional values (such as The Philippines) had higher BGSD than MDMW (Figure 6E). 

The relationship between IDISW (and COV) and TI is also non-linear (Figure 6F). The range of IDISW and TI values across countries was much wider for zero-dose than for DTP3 coverage, showing increased sensitivity to small numbers in the zero-dose indicator. For example, Turkmenistan (MICS 2015) had subnational zero-dose prevalence ranging from 0 to 3%, causing high TI and IDISW values of 110 and 992, respectively. IDISW and TI are both sensitive to indicator estimates that are further from the setting average. Costa Rica (MICS 2018) had a single region with zero-dose prevalence of 14%, while all other regions had a prevalence of 7% or less, leading to high IDISW and TI values. 

### 3.4. Subnational Inequality Ordered by HDI

When subnational regions were ordered by their HDI values, patterns of inequality in DTP3 and zero-dose immunization and correlations across measures were overall similar to those observed with economic-related inequality and are therefore not reported here. All country-level results are available in Supplementary Table S1. 

### 3.5. Quantifying the Impact of Addressing Inequality

**Figure 7:**
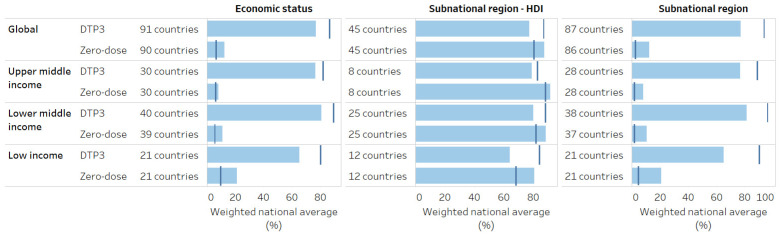
Potential improvement in the national average by eliminating inequality (2013–2022). Note: The potential for improvement (dark blue vertical line) represents the overall weighted average that would be possible if, in each country, the whole population had the same level of coverage as the most advantaged subgroup. The current weighted average is indicated by the light blue bar.

Figure 7 illustrates the impact of eliminating inequality in DTP3 and zero-dose prevalence. For example, for DTP3 coverage, the overall weighted national average across 91 countries is 77%. If all countries improved the level of coverage to that of the richest subgroup, the overall weighted national average would increase to 86%. Without economic-related inequality, zero-dose prevalence would halve, decreasing overall from 12% to 6%. DTP3 coverage would increase by 16 percentage points, and zero-dose prevalence would decrease by 10 percentage points if there was no subnational inequality in all study countries. Potential improvement was largest in low-income countries. 

PAR and PAF were strongly correlated with other measures for DTP3 coverage (Table 5). For instance, when there were high absolute economic inequalities in DTP3 coverage, there was also a high potential for improvement in both absolute and relative terms. For zero-dose, patterns were more mixed. For economic status, PAR was more strongly correlated with other relative measures of inequality, while PAF tended to be more correlated with absolute measures. For subnational regions ordered by HDI, PAR had moderate correlations with other measures, indicating that in some countries, high subnational inequality related to HDI did not translate to a high potential for improvement. This usually occurred in contexts where the subnational region with the highest HDI was not the region with the highest DTP3 coverage or lowest zero-dose prevalence (e.g., Chad) and/or was a region with small population size (e.g., Ethiopia). 

## 4. Discussion

Using data from 92 countries, we compared inequalities in DTP3 and zero-dose immunization indicators related to place of residence, economic status, and subnational region, using a total of 16 summary measures. The 16 measures showed more commonalities than differences overall. Nevertheless, an in-depth analysis of general patterns, correlations, and specific country cases indicates that the selection of measures can influence the assessment of inequality. 

### 4.1. Absolute vs. Relative Measures

Absolute and relative measures were not always strongly correlated and, in some cases, showed different results in terms of immunization inequalities. For instance, there were absolute inequalities in DTP3 coverage related to place of residence and economic status that overall favored urban areas and the richest, but inequalities were low in relative terms. Subnational inequality was overall higher for DTP3 coverage than zero-dose in absolute terms, but the opposite was true in relative terms. 

When measured in relative terms, there was a larger variation across countries in the magnitude of inequality in zero-dose prevalence related to place of residence, economic status, and ordered subnational regions compared to DTP3 coverage. This shows that relative measures tend to be more sensitive to small numbers because when the denominator in a ratio is small, any change in the numerator leads to a large relative change (while the corresponding absolute change will be small) [25]. Therefore, this stresses the importance of using both absolute and relative measures alongside each other, as they each capture a different aspect of inequality. This also reflects a normative judgment about the importance placed on inequality per se [26]. Most absolute measures can be transformed into relative measures, and vice versa; the WHO ‘healthequal’ packages include both types of measures. 

### 4.2. Simple vs. Complex Measures

Absolute and relative inequality in DTP3 coverage across wealth quintiles and across subnational regions ordered by HDI produced similar conclusions overall when measured using simple and complex measures. However, there was a low correlation between subnational inequality measured using simple measures and complex measures for the zero-dose indicator. At the country level, we encountered cases of simple and complex measures showing different patterns of inequality due to simple measures not taking all subgroups and population sizes into account. 

The results of the analysis also showed that using differences and ratios could produce higher magnitudes of inequality when influenced by outlier subgroups (subgroups with exceptionally small or large indicator values), a phenomenon that was moderated by using a weighted, complex measure. Heterogeneity naturally increases with a larger number of subgroups; therefore, difference and ratio may be better adequate when applied to a small number of subgroups. Moreover, simple measures may be useful when comparing indicator values of a non-ordered dimension, such as occupation or ethnicity, when the aim is to compare two specific subgroups without making assumptions about a hierarchical structure.

The choice between reporting simple or complex measures depends on the underlying data and the target audience. The strengths of simple measures are their mathematical simplicity and ease of understanding; the weaknesses are that they are only based on two data points without taking the sizes of the groups into account. In cases where simple and complex measures have similar results, reporting a simple measure of inequality may be preferable, particularly when the target audience is general and non-technical. A complex measure may be applied if simple and complex measures do not have the same results and/or if attempting to use a more rigorous method that takes population subgroup sizes and the effect size of all subgroups into account. Complex measures are more complicated to calculate than simple measures and often require statistical knowledge—for instance, the calculation of SII and RII requires the use of a regression model and the knowledge to choose the best model. However, the WHO ‘healthequal’ Stata and R packages facilitate the process of calculating complex measures (and their confidence intervals, where possible). 

### 4.3. Choice of Complex Measure for Ordered Dimensions

In this paper, we explored inequalities across socioeconomic groups, using measures of economic status at the household level (wealth quintiles) and area-based measures at the regional level (subnational HDI). Area-based measures have an advantage in informing place-based decision-making [27]. The summary measures SII, RII, ACI, and RCI are recommended for use with ordered dimensions of inequality since they show directionality in addition to the magnitude of inequality, helping to identify the disadvantaged subgroup [11]. 

Using absolute complex measures (ACI and SII), inequality in DTP3 and zero-dose tended to favor the richest quintile and subnational regions with higher HDI. However, in some countries, the opposite is true. ACI and SII provide almost identical measures of inequality since there is a close mathematical correspondence between the two. Therefore, the choice between these measures depends on the interpretation requirements. SII is generally easier to communicate than ACI since it has the same interpretation as difference. Similarly, RII is easier to interpret than RCI. The interpretation of ACI and RCI tends to be less straightforward since they describe the extent of concentration of an indicator among most- or least-advantaged populations. An alternative calculation of the concentration index, which multiplies the index by 75, conceptualizes it in terms of the percentage of a variable that needs to be redistributed (e.g., from richer to poorer segments of the population) to achieve perfect equality [28]. While this may facilitate understanding by non-technical audiences, it may not be useful when applied to immunization coverage measures as coverage cannot be ‘redistributed’. 

### 4.4. Choice of Complex Measure for Non-Ordered Dimensions

We used the subnational region as an example of a non-ordered dimension of inequality, meaning that subgroups (regions) have no inherent ranking. Other examples of non-ordered dimensions include ethnicity, occupation, religion, and marital status. The results demonstrated that comparing inequality using non-ordered dimensions that are composed of different numbers of subgroups can be challenging—a greater number of subgroups can increase the level of heterogeneity, while a small number of regions can decrease the level of heterogeneity (a resolution issue) [27]. When monitoring subnational inequality, a potential method to control for this is to group subnational regions into quartiles or quintiles such that there is an equal number of data points for each country; however, this only works when the number of subnational regions is sufficient. 

Subnational inequality in immunization measured using the weighted and unweighted mean difference from mean was highly correlated, though the weighted measures produced a lower magnitude of inequality in situations when outlier regions had small population sizes. The choice of weighted versus unweighted measure is a value judgement and depends on the research aim [26]. For example, one might argue that subnational regions have normative importance regardless of their size and, thus, should be treated equally. On the other hand, one might argue that the immunization coverage of all individuals within regions is important; therefore, individuals should be weighted equally (regions should be weighted by their population size). 

In addition to choices of absolute versus relative and weighted versus unweighted, the choice of complex measure of immunization inequality related to non-ordered dimensions also depends on the target audience, interpretation, and the importance of sensitivity to extreme or outlier values. The mean difference from mean (MDMW), variance (BGV), and standard deviation (BGSD) are measures of dispersion that may be familiar and intuitive to non-technical audiences. MDMW and BGSD values are limited to the scale of the indicator (e.g., 100 in terms of indicators measured as a percentage). In contrast, BGV, COV, and TI have infinity scales, which may pose challenges in terms of interpreting and judging situations of high inequality (e.g., setting an absolute threshold for ‘high inequality’ in childhood immunization). 

The results show that BGV, BGSD, and TI are measures of inequality that are more sensitive to the presence of outlier values and, therefore, may capture when certain population subgroups are being left behind the others. BGV has such sensitivity due to squaring differences from the mean. BGSD, a transformation of BGV, retains this sensitivity but takes the unit of the indicator, facilitating its interpretation in comparison to BGV, which is measured in squared units. TI produces a number that reflects the extent to which the distribution of the indicator across groups differs from the distribution of the population across those same groups. It is more difficult to explain and interpret to non-technical audiences—but there are advantages in its increased sensitivity to top-end inequality (e.g., subnational regions with higher immunization coverage) and its potential use to decompose inequality across groups [29]. 

### 4.5. Measuring the Impact of Eliminating Inequality

We found that if all countries brought the level of immunization coverage to that of the richest subgroup, the overall average across 92 low- and middle-income countries would increase to 86%, and zero-dose prevalence would reduce to 6% (from 77% and 12%, respectively). PAR and PAF take into account the size of the population because the larger the size of the subgroups with low immunization coverage, the greater the potential improvement in the overall average. 

Measuring inequality using PAR/PAF is particularly powerful from a decision-maker’s perspective because it shows how much can be achieved by eliminating inequality. They are, therefore, an ideal complement to other measures of inequality and are suitable in situations where the purpose is to communicate information for rapid decision-making. Using PAR, it is also possible to calculate the magnitude of the reduction in inequality needed in each subgroup to achieve full equality, a measurement that is useful for decision-makers because it allows the estimation of immunization targets [30].

### 4.6. Limitations

Household survey data, while useful for monitoring inequalities in immunization, have several limitations. Surveys are not flawless, often suffering from sampling bias, where certain populations may be underrepresented. Children missing vaccination cards may cause caregivers to misreport vaccination histories. In addition, small sample sizes in specific subgroups can limit the precision of estimates. Moreover, the data analyzed in this study are from different years across countries, making direct comparisons challenging, and not all countries conduct these surveys, resulting in gaps in global coverage monitoring.

## 5. Conclusions

The pursuit of equity is a central tenant of the Immunization Agenda 2030 and the Sustainable Development Agenda. Measuring immunization inequalities is the first step in making decisions that implement actions and strategies aimed at reducing and eliminating them. Once such interventions have been implemented, it is also important to monitor their impact. 

Summary measures of inequality are useful in that they yield a single number to assess immunization inequality over time, across indicators, and across settings. The selection of appropriate summary measures to monitor inequalities entails consideration of underlying assumptions and value judgement related to absolute versus relative measures, simple versus complex measures, weighted versus unweighted measures, sensitivity to outliers, target audience, and interpretation. Such assumptions and value judgements should be made explicit in the research. Conducting immunization inequality monitoring also requires an adequate health information base, with regular data collection incorporating dimensions of inequality, analytical capacities, and responsive and accountable processes to support equity-oriented actions. 

## Figures and Tables

**Table 1:** Number of countries with data included in the analysis for each immunization indicator and inequality dimension.

Immunization Indicator	Place of Residence	Economic Status	Subnational Region	Subnational Region Ordered by HDI
DTP3 coverage	92	91	87	45
Zero-dose prevalence	91	90	86	45

HDI: Human Development Index.

**Table 2:** Summary measures of health inequality are calculated in the analysis for each dimension of inequality.

Measure Type	Measure Name	Absolute or Relative	Dimension of Inequality
Simple measures	Difference (D)	Absolute	Place of residence Economic status Subnational region Subnational region ordered by HDI
	Ratio (R)	Relative	Place of residence Economic status Subnational region Subnational region ordered by HDI
Disproportionality measures (ordered dimensions)	Absolute concentration index (ACI)	Absolute	Economic status Subnational region ordered by HDI
	Relative concentration index (RCI)	Relative	Economic status Subnational region ordered by HDI
Regression-based measures (ordered dimensions)	Slope index of inequality (SII)	Absolute	Economic status Subnational region ordered by HDI
	Relative index of inequality (RII)	Relative	Economic status Subnational region ordered by HDI
Variance measures (non-ordered dimensions)	Between-group variance (BGV)	Absolute	Subnational region
	Between-group standard deviation (BGSD)	Absolute	Subnational region
	Coefficient of variation (COV)	Relative	Subnational region
Mean difference measures (non-ordered dimensions)	Unweighted mean difference from mean (MDMU)	Absolute	Subnational region
	Weighted mean difference from mean (MDMW)	Absolute	Subnational region
	Unweighted index of disparity (IDISU)	Relative	Subnational region
	Weighted index of disparity (IDISW)	Relative	Subnational region
Disproportionality measures (non-ordered dimensions)	Theil index (TI)	Relative	Subnational region
Impact measures	Population attributable risk (PAR)	Absolute	Economic status Subnational region Subnational region ordered by HDI
	Population attributable fraction (PAF)	Relative	Economic status Subnational region Subnational region ordered by HDI

**Table 3:** Economic status: Correlations between summary measures (2013–2022).

Indicator	Measure	D	R	ACI	RCI	SII	RII
DTP3 coverage	D	1					
	R	0.9913 **	1				
	ACI	0.9799 **	0.9742 **	1			
	RCI	0.9727 **	0.9827 **	0.9899 **	1		
	SII	0.9790 **	0.9725 **	0.9993 **	0.9881 **	1	
	RII	0.9738 **	0.9824 **	0.9908 **	0.9990 **	0.9902 **	1
Zero-dose prevalence	D	1					
	R	0.6523 **	1				
	ACI	0.9654 **	0.6037 **	1			
	RCI	0.6743 **	0.8247 **	0.6993 **	1		
	SII	0.9656 **	0.6098 **	0.9997 **	0.7061 **	1	
	RII	0.6770 **	0.8248 **	0.7022 **	0.9998 **	0.7090 **	1

D: difference; R: ratio; ACI: absolute concentration index; RCI: relative concentration index; SII: slope index of inequality; RII: relative index of inequality; ** *p* < 0.01; Dark green: r_s_ ≥ 0.9; Light green: r_s_ ≥ 0.7; Orange: r_s_ ≥ 0.4.

**Table 4:** Subnational region: Correlations between summary measures (2013–2022).

		D	R	MDMU	MDMW	IDISU	IDISW	BGV	BGSD	COV	TI
DTP3 coverage	D	1									
	R	0.9819 **	1								
	MDMU	0.9250 **	0.9311 **	1							
	MDMW	0.8877 **	0.8891 **	0.9468 **	1						
	IDISU	0.9063 **	0.9435 **	0.9773 **	0.9400 **	1					
	IDISW	0.8806 **	0.9101 **	0.9427 **	0.9824 **	0.9690 **	1				
	BGV	0.9329 **	0.9294 **	0.9579 **	0.9861 **	0.9462 **	0.9722 **	1			
	BGSD	0.9329 **	0.9294 **	0.9579 **	0.9861 **	0.9462 **	0.9722 **	1.0000 **	1		
	COV	0.9093 **	0.9395 **	0.9488 **	0.9709 **	0.9770 **	0.9923 **	0.9770 **	0.9770 **	1	
	TI	0.9156 **	0.9459 **	0.9505 **	0.9678 **	0.9779 **	0.9902 **	0.9772 **	0.9772 **	0.9992 **	1
Zero-dose prevalence	D	1									
	R	0.3700 **	1								
	MDMU	0.9695 **	0.2875 *	1							
	MDMW	0.9155 **	0.2368	0.9488 **	1						
	IDISU	0.3864 **	0.6732 **	0.3420 **	0.2548	1					
	IDISW	0.3096 *	0.5625 **	0.2944 *	0.3944 **	0.7694 **	1				
	BGV	0.9542 **	0.2888 *	0.9688 **	0.9858 **	0.3122 *	0.3840 **	1			
	BGSD	0.9542 **	0.2888 *	0.9688 **	0.9858 **	0.3122 *	0.3840 **	1.0000 **	1		
	COV	0.3134 *	0.6695 **	0.2510	0.3092 *	0.8462 **	0.9390 **	0.3369 **	0.3369 **	1	
	TI	0.3147 *	0.7086 **	0.2759 *	0.3513 **	0.8071 **	0.9590 **	0.3642 **	0.3642 **	0.9747 **	1

D: difference; R: ratio; MDMU: unweighted mean difference from mean; MDMW: weighted mean difference from mean; IDISU: unweighted index of disparity; IDISW: weighted index of disparity; BGV: between-group variance; BGSD: between-group standard deviation; COV: coefficient of variation; TI: Theil index. * *p* < 0.05; ** *p* < 0.01; Dark green: r_s_ ≥ 0.9; Light green: r_s_ ≥ 0.7; Orange: r_s_ ≥ 0.4; Yellow: r_s_ < 0.4.

**Table 5:** Correlations between PAR, PAF, and other summary measures (2013–2022).

	DTP3 Coverage						Zero-Dose Prevalence					
	Economic Status		Subnational Region Ordered by HDI		Subnational Region		Economic Status		Subnational Region Ordered by HDI		Subnational Region	
	PAR	PAF	PAR	PAF	PAR	PAF	PAR	PAF	PAR	PAF	PAR	PAF
D	0.9427 **	0.9424 **	0.7673 **	0.7704 **	0.8739 **	0.8905 **	0.6214 **	0.9152 **	0.7038 **	0.8054 **	−0.2180	−0.9093 **
R	0.9325 **	0.9238 **	0.8072 **	0.7992 **	0.9025 **	0.8954 **	0.9517 **	0.6984 **	0.8097 **	0.6080 **	−0.8882 **	−0.2957 *
ACI	0.9063 **	0.9060 **	0.7689 **	0.7565 **			0.5506 **	0.8554 **	0.6565 **	0.8313 **		
RCI	0.9052 **	0.8973 **	0.7951 **	0.7797 **			0.7046 **	0.5959 **	0.6547 **	0.4776 **		
SII	0.9056 **	0.9056 **	0.7695 **	0.7569 **			0.5573 **	0.8567 **	0.6620 **	0.8204 **		
RII	0.9075 **	0.9003 **	0.7928 **	0.7776 **			0.7051 **	0.5977 **	0.6529 **	0.4818 **		
MDMU					0.8873 **	0.8932 **					−0.1798	−0.9346 **
MDMW					0.9090 **	0.9181 **					−0.1835	−0.9236 **
IDISU					0.9247 **	0.9081 **					−0.4185 **	−0.1365
IDISW					0.9389 **	0.9268 **					−0.4456 **	−0.179
BGV					0.9097 **	0.9215 **					−0.2073	−0.9323 **
BGSD					0.9097 **	0.9215 **					−0.2073	−0.9323 **
COV					0.9422 **	0.9289 **					−0.4805 **	−0.1238
TI					0.9382 **	0.9244 **					−0.5796 **	−0.1879

D: difference; R: ratio; ACI: absolute concentration index; RCI: relative concentration index; SII: slope index of inequality; RII: relative index of inequality; MDMU: unweighted mean difference from mean; MDMW: weighted mean difference from mean; IDISU: unweighted index of disparity; IDISW: weighted index of disparity; BGV: between-group variance; BGSD: between-group standard deviation; COV: coefficient of variation; TI: Theil index; HDI: Human Development Index. * *p* < 0.05; ** *p* < 0.01; Dark green: r_s_ ≥ 0.9; Light green: r_s_ ≥ 0.7; Orange: r_s_ ≥ 0.4; Yellow: r_s_ < 0.4.

## Data Availability

All analyses were carried out using publicly available datasets that can be obtained from the WHO Health Inequality Data Repository (https://www.who.int/data/inequality-monitor/data, accessed 13 September 2024). Analyses used the latest available dataset versions as of 13 September 2024.

## Supplementary Materials

The following supporting information can be downloaded at https://www.mdpi.com/article/10.3390/vaccines12121324/s1, Supplementary File S1: Formulas for the calculation of summary measures of health inequality, refs. [5,31,32,33,34,35] are cited in File S1; Table S1: Country-level summary measure results.

## References

[1] World Health Organization (2013). Handbook on Health Inequality Monitoring: With a Special Focus on Low- and Middle-Income Countries.

[2] Farrenkopf B.A., Zhou X., Shet A., Olayinka F., Carr K., Patenaude B., Chido-Amajuoyi O.G., Wonodi C. (2023). Understanding Household-Level Risk Factors for Zero Dose Immunization in 82 Low- and Middle-Income Countries. PLoS ONE.

[3] Oyo-Ita A., Oduwole O., Arikpo D., Effa E.E., Esu E.B., Balakrishna Y., Chibuzor M.T., Oringanje C.M., Nwachukwu C.E., Wiysonge C.S. (2023). Interventions for Improving Coverage of Childhood Immunisation in Low- and Middle-Income Countries. Cochrane Database Syst. Rev..

[4] World Health Organization Immunization Agenda 2030: A Global Strategy to Leave No One Behind. https://www.who.int/publications/m/item/immunization-agenda-2030-a-global-strategy-to-leave-no-one-behind.

[5] Schlotheuber A., Hosseinpoor A.R. (2022). Summary Measures of Health Inequality: A Review of Existing Measures and Their Application. Int. J. Environ. Res. Public Health.

[6] Cata-Preta B.O., Wehrmeister F.C., Santos T.M., Barros A.J.D., Victora C.G. (2021). Patterns in Wealth-related Inequalities in 86 Low- and Middle-Income Countries: Global Evidence on the Emergence of Vaccine Hesitancy. Am. J. Prev. Med..

[7] Arsenault C., Harper S., Nandi A., Mendoza Rodríguez J.M., Hansen P.M., Johri M. (2017). Monitoring Equity in Vaccination Coverage: A Systematic Analysis of Demographic and Health Surveys from 45 Gavi-Supported Countries. Vaccine.

[8] Johns N.E., Blumenberg C., Kirkby K., Allorant A., Costa F.D.S., Danovaro-Holliday M.C., Lyons C., Yusuf N., Barros A.J.D., Hosseinpoor A.R. (2024). Comparison of Wealth-Related Inequality in Tetanus Vaccination Coverage before and during Pregnancy: A Cross-Sectional Analysis of 72 Low- and Middle-Income Countries. Vaccines.

[9] Lyons C., Nambiar D., Johns N.E., Allorant A., Bergen N., Hosseinpoor A.R. (2024). Inequality in Childhood Immunization Coverage: A Scoping Review of Data Sources, Analyses, and Reporting Methods. Vaccines.

[10] Wagstaff A., Paci P., van Doorslaer E. (1991). On the Measurement of Inequalities in Health. Soc. Sci. Med..

[11] Mackenbach J.P., Kunst A.E. (1997). Measuring the Magnitude of Socio-Economic Inequalities in Health: An Overview of Available Measures Illustrated with Two Examples from Europe. Soc. Sci. Med..

[12] Harper S., Lynch J., Reichman M.E., Reeve B., Breen N. (2005). Selected Comparisons of Measures of Health Disparities.

[13] The R Foundation R: The R Project for Statistical Computing. https://www.r-project.org/.

[14] StataCorp Stata: Statistical Software for Data Science. https://www.stata.com/.

[15] Kirkby K., Schlotheuber A., Vidal Fuertes C., Ross Z., Hosseinpoor A.R. (2022). Health Equity Assessment Toolkit (HEAT and HEAT Plus): Exploring Inequalities in the COVID-19 Pandemic Era. Int. J. Equity Health.

[16] Corsi D.J., Neuman M., Finlay J.E., Subramanian S.V. (2012). Demographic and Health Surveys: A Profile. Int. J. Epidemiol..

[17] Khan S., Hancioglu A. (2019). Multiple Indicator Cluster Surveys: Delivering Robust Data on Children and Women across the Globe. Stud. Fam. Plan..

[18] World Health Organization Health Inequality Data Repository. https://www.who.int/data/inequality-monitor/data.

[19] Global Data Lab Area Database. https://globaldatalab.org/areadata/.

[20] Smits J., Permanyer I. (2019). The Subnational Human Development Database. Sci. Data.

[21] Filmer D., Pritchett L.H. (2001). Estimating Wealth Effects without Expenditure Data—Or Tears: An Application to Educational Enrollments in States of India. Demography.

[22] O’Donnell O., Van Doorslaer D.E., Wagstaff A., Lindelow M. (2008). Analyzing Health Equity Using Household Survey Data a Guide to Techniques and Their Implementation.

[23] The World Bank World Bank Country and Lending Groups. https://datahelpdesk.worldbank.org/knowledgebase/articles/906519-world-bank-country-and-lending-groups.

[24] Schober P., Schwarte L.A. (2018). Correlation Coefficients: Appropriate Use and Interpretation. Anesth. Analg..

[25] Asada Y. (2010). On the Choice of Absolute or Relative Inequality Measures. Milbank Q..

[26] Harper S., King N.B., Meersman S.C., Reichman M.E., Breen N., Lynch J. (2010). Implicit Value Judgments in the Measurement of Health Inequalities. Milbank Q..

[27] Hosseinpoor A.R., Bergen N. (2016). Area-Based Units of Analysis for Strengthening Health Inequality Monitoring. Bull. World Health Organ..

[28] Koolman X., van Doorslaer E. (2004). On the Interpretation of a Concentration Index of Inequality. Health Econ..

[29] Conceicao P.N., Ferreira P.M. (2000). The Young Person’s Guide to the Theil Index: Suggesting Intuitive Interpretations and Exploring Analytical Applications. UTIP Working Paper No. 14. https://ssrn.com/abstract=228703.

[30] Schneider M.C., Castillo-Salgado C., Bacallao J., Loyola E., Mujica O.J., Vidaurre M., Roca A. (2002). Methods for Measuring Inequalities in Health. Rev. Panam. Salud Publica/Pan Am. J. Public Health.

[31] Ahn J., Harper S., Yu M., Feuer E.J., Liu B., Luta G. (2018). Variance Estimation and Confidence Intervals for 11 Commonly Used Health Disparity Measures. JCO Clin. Cancer Inform..

[32] Kakwani N.C. (1980). Income inequality and poverty: Methods of estimation and policy applications. Popul. Dev. Rev..

[33] Ahn J., Harper S., Yu M., Feuer E.J., Liu B. (2019). Improved Monte Carlo methods for estimating confidence intervals for eleven commonly used health disparity measures. PLoS ONE.

[34] Pearcy J.N., Keppel K.G. (2002). A summary measure of health disparity. Public Health Rep..

[35] Walter S.D. (1978). Calculation of Attributable Risks from Epidemiological Data. Int. J. Epidemiol..

