# Supplementary material for: Quantifying Inequalities in Childhood Immunization Using Summary Measures of Health Inequality: An Application of WHO Stata and R ‘Healthequal’ Packages
Source: Vaccines (Basel). 2024 Nov 26;12(12):1324. doi: 10.3390/vaccines12121324 (PMC11680256; doi:10.3390/vaccines12121324)
Supplement: Supplementary file 1 [file vaccines-12-01324-s001.zip › Supplementary File 1.pdf]

Supplementary File S1: Formulas for the calculation of summary measures of health inequality

This document contains the calculation methodologies for 21 summary measures of health inequality and their variances, using disaggregated data as inputs. The notation for the formulas is described in Table S2.

Table S2. Notation for formulas

y_j	Indicator value for subgroup *j*
σ_j	Standard error of the indicator value for subgroup *j*
p_j	Population share of subgroup *j* out of the total population
X_j	Relative rank of subgroup *j*, calculated as $\sum_{i=1}^{j} p_i - 0.5p_j$
$\bar{X}$	Weighted average rank, calculated as $\sum_j p_j X_j$
μ	Weighted average indicator value (e.g. national average)
$\hat{v}_0$	Predicted value of the hypothetical person at the bottom of the social-group distribution (rank 0)
$\hat{v}_1$	Predicted value of the hypothetical person at the top of the social-group distribution (rank 1)
n	Number of subgroups
y_{ref}	Indicator value for a reference point

Simple measures

Difference

Difference (D) is an absolute measure of inequality that shows the difference in an indicator between two population subgroups. The selection of the two subgroups depends on the characteristics of the inequality dimension, the type of indicator (favourable or adverse), and the purpose of the analysis (1). D has the same unit as the indicator. Greater absolute values indicate higher levels of inequality; D is zero if there is no inequality.

D is calculated as:

$$D = y_1 - y_2$$

The variance of D is calculated as (2):

$$var(D) = {\sigma_1}^2 + {\sigma_2}^2$$

Ratio

Ratio (R) is a relative measure of inequality that shows the ratio of an indicator between two population subgroups. The selection of the two subgroups depends on the characteristics of the inequality dimension, the type of indicator (favourable or adverse), and the purpose of the analysis (1).

R has no unit and only assumes positive values. The further the value of R from one, the higher the level of inequality; R is one if there is no inequality.

R is calculated as:

$$R = \frac{y_1}{y_2}$$

The variance of R is calculated as (2):

$$var(R) = \left(\frac{1}{y_2}\right)^2 {\sigma_1}^2 + \left(\frac{y_1}{y_2}\right)^2 {\sigma_2}^2$$

Disproportionality measures (for ordered dimensions)

Absolute concentration index

The absolute concentration index (ACI) is an absolute measure of inequality that indicates the extent to which an indicator is concentrated among disadvantaged or advantaged subgroups, on an absolute scale. ACI has the same unit as the indicator. The larger the absolute value of ACI, the higher the level of inequality. Positive values indicate a concentration of the indicator among advantaged subgroups, and negative values indicate a concentration of the indicator among disadvantaged subgroups. ACI is zero if there is no inequality.

ACI is calculated as:

$$ACI = \sum_j p_j (2X_j - 1)\, y_j$$

The variance of ACI is calculated as (2):

$$var(ACI) = \sum_j {p_j}^2 {\sigma_j}^2 (2X_j - 1)^2$$

Relative concentration index

The relative concentration index (RCI) is a relative measure of inequality that indicates the extent to which an indicator is concentrated among disadvantaged or advantaged subgroups, on a relative scale (3). RCI has no unit. The larger the absolute value of RCI, the higher the level of inequality. Positive values indicate a concentration of the indicator among advantaged subgroups, and negative values indicate a concentration of the indicator among disadvantaged subgroups. RCI is zero if there is no inequality.

RCI is calculated as:

$$RCI = \frac{\sum_j p_j (2X_j - 1)\, y_j}{\mu}$$

The variance of RCI is calculated as (2):

$$var(RCI) = \frac{\sum_j p_j{}^2 \sigma_j{}^2 \left[\left(2X_j - 1\right) - RCI\right]^2}{\mu^2}$$

Regression-based measures (for ordered dimensions)

Slope index of inequality

The slope index of inequality (SII) is an absolute measure of inequality that represents the difference in predicted values of an indicator between the most advantaged and most disadvantaged subgroups, obtained by fitting a regression model. SII has the same unit as the indicator. SII is zero if there is no inequality. Greater absolute values indicate higher levels of inequality. Positive values indicate that the level of the indicator is higher among advantaged subgroups, while negative values indicate that the level of the indicator is higher among disadvantaged subgroups. Note that this results in different interpretations for favourable and adverse indicators.

To calculate SII, a weighted sample of the whole population is ranked from the most disadvantaged subgroup (at rank 0) to the most advantaged subgroup (at rank 1). The indicator of interest is then regressed against this relative rank (X_j) using an appropriate regression model (such as a generalized linear model with logit link, which bounds estimates between 0 and 1 and is therefore more accurate for indicators measured as percentages). The difference between the predicted values of the indicator at the two extremes ($\hat{v}_1$ and $\hat{v}_0$) is then calculated:

$$SII = \hat{v}_1 - \hat{v}_0$$

The variance of SII is estimated from the regression model using the delta method (2).

Relative index of inequality

The relative index of inequality (RII) is a relative measure of inequality that represents the ratio of the predicted values of an indicator between the most advantaged and most disadvantaged, obtained by fitting a regression model. RII has no unit and takes only positive values. RII has the value of 1 if there is no inequality. Values larger than 1 indicate that the level of the indicator is higher among advantaged subgroups, and values lower than 1 indicate that the level of the indicator is higher among disadvantaged subgroups. Note that this results in different interpretations for favourable and adverse indicators.

To calculate RII, a weighted sample of the whole population is ranked from the most disadvantaged subgroup (at rank 0) to the most advantaged subgroup (at rank 1). The indicator of interest is then regressed against this relative rank (X_j) using an appropriate regression model (such as a generalized linear model with logit link, which bounds estimates between 0 and 1 and is therefore more accurate for indicators measured as percentages). The ratio of the predicted values of the indicator at the two extremes ($\hat{v}_1$ and $\hat{v}_0$) is then calculated:

$$RII = \hat{v}_1 \,/\, \hat{v}_0$$

The variance of RII is estimated from the regression model using the delta method (2)[1] based on a logarithmic version of RII, which is then exponentiated so that confidence intervals are non-symmetrical (since RII is measured on a logarithmic scale).

Variance measures (for non-ordered dimensions)

Between-group variance

Between-group variance (BGV) is an absolute measure of inequality that considers all population subgroups. Subgroups are weighted according to their population share. BGV is reported as the squared unit of the indicator. BGV has only positive values, with larger values indicating higher levels of inequality. BGV is zero if there is no inequality. BVG is calculated as the weighted average of squared differences between the subgroup estimates and the setting average, weighted by each subgroup's population share:

$$BGV = \sum_j p_j (y_j - \mu)^2$$

The variance of BGV is calculated as (2):

$$var(BGV) = 4\sum_j {p_j}^2 {\sigma_j}^2 \, (y_j - \mu)^2 + 2\left[\left(\sum_j {p_j}^2 {\sigma_j}^2\right)^2 - \left(\sum_j {p_j}^4 {\sigma_j}^4\right) + \left(\sum_j {p_j}^2 \left(1 - p_j\right)^2 {\sigma_j}^4\right)\right]$$

Between-group standard deviation

Between-group standard deviation (BGSD) is an absolute measure of inequality that considers all population subgroups. Subgroups are weighted according to their population share. BGSD is reported in the unit of the indicator. BGSD has only positive values, with larger values indicating higher levels of inequality. BGSD is zero if there is no inequality. BGSD is calculated as the square root of BGV:

$$BGSD = \sqrt{\sum_j p_j (y_j - \mu)^2}$$

95% confidence intervals of BGSD are calculated using a Monte Carlo simulation-based method (4). The dataset is simulated 100 times, with the mean and standard error of each simulated dataset being the same as the original dataset. BGSD is calculated for each of the simulated sample datasets. The 95% confidence intervals are based on the 2.5th and 97.5th centiles of the BGSD results.

Coefficient of variation

Coefficient of variation (COV) is a relative measure of inequality that considers all population subgroups. Subgroups are weighted according to their population share. COV has no unit. COV has only positive

[1] RII is called the Kunst-Mackenbach relative index (KMI) in (2).

values, with larger values indicating higher levels of inequality. COV is zero if there is no inequality. COV is calculated as BGSD divided by the setting average, multiplied by 100:

$$COV = \frac{\sqrt{\sum_j p_j (y_j - \mu)^2}}{\mu} * 100$$

95% confidence intervals of COV are calculated using the same method as for BGSD.

Mean difference measures (for non-ordered dimensions)

Mean difference from best-performing subgroup

Mean difference from best-performing subgroup (MDB) is an absolute measure of inequality that shows the mean difference between each population subgroup and the best-performing subgroup (y_{ref}). y_{ref} is the subgroup with the highest value in the case of favorable indicators and the subgroup with the lowest value in the case of adverse indicators. MDB has the same unit as the indicator. MDBU is unweighted, where all subgroups are weighted equally. MDBW is weighted, where subgroups are weighted according to their population share. MDBU and MDBW only have positive values, with larger values indicating higher levels of inequality. MDBU and MDBW are zero if there is no inequality.

MDBU is calculated as:

$$MDBU = \frac{1}{n} * \sum_j |y_j - y_{ref}|$$

MDBW is calculated as:

$$MDBW = \sum_j p_j |y_j - y_{ref}|$$

95% confidence intervals of MDBU and MDBW are calculated using the same method as for BGSD.

Mean difference from mean

Mean difference from mean (MDM) is an absolute measure of inequality that shows the mean difference between each population subgroup and the mean (e.g. the national average). MDM has the same unit as the indicator. MDMU is unweighted, where all subgroups are weighted equally. MDMW is weighted, where subgroups are weighted according to their population share. MDMU and MDMW only have positive values, with larger values indicating higher levels of inequality. MDMU and MDMW are zero if there is no inequality.

MDMU is calculated as:

$$MDMU = \frac{1}{n} * \sum_j |y_j - \mu|$$

MDMW is calculated as:

$$MDMW = \sum_j p_j |y_j - \mu|$$

95% confidence intervals of MDMU and MDMW are calculated using the same method as for BGSD.

Mean difference from a reference point

Mean difference from a reference point (MDR) is an absolute measure of inequality that shows the mean difference between each population subgroup and a defined reference subgroup (e.g. the capital city or region for data disaggregated by subnational regions). MDR has the same unit as the indicator. MDRU is unweighted, where all subgroups are weighted equally. MDRW is weighted, where subgroups are weighted according to their population share. MDRU and MDRW and their confidence intervals are calculated using the same method as MDBU and MDBW, with y_{ref} being the subgroup estimate of the reference subgroup.

95% confidence intervals of MDRU and MDRW are calculated using the same method as for BGSD.

Index of disparity

The index of disparity (IDIS) is a relative measure of inequality that shows the mean difference between each subgroup and the weighted average, on a relative scale (5). IDIS has no unit. IDISU is unweighted, where all subgroups are weighted equally. IDISW is weighted, where subgroups are weighted according to their population share. IDISU and IDISW have only positive values, with larger values indicating higher levels of inequality. IDISU and IDISW are zero if there is no inequality.

IDISU is calculated as:

$$IDISU = \frac{\frac{1}{n} * \sum_j |y_j - \mu|}{\mu} * 100$$

IDISW is calculated as:

$$IDISW = \frac{\sum_j p_j \, |y_j - \mu|}{\mu} * 100$$

95% confidence intervals of IDISU and IDISW are calculated using the same method as for BGSD.

Disproportionality measures (for non-ordered dimensions)

Theil index

The Theil index (TI) is a relative measure of inequality that considers all population subgroups. Subgroups are weighted according to their population share. TI measures the extent to which the shares of the population and shares of the health indicator differ across subgroups, weighted by shares of the health

indicator. TI has no unit. It may be more easily interpreted when multiplied by 1000. Greater absolute values indicate higher levels of inequality. TI is zero if there is no inequality. TI is calculated as:

$$TI = \sum_j p_j \frac{y_j}{\mu} \ln \frac{y_j}{\mu} * 1000$$

The variance of TI is calculated as (2):

$$var(TI) = \frac{\sum_j p_j{}^2 \sigma_j{}^2 \left[\left\{1 + \ln\left(\frac{y_j}{\mu}\right)\right\} - \left\{\sum_k p_k \frac{y_k}{\mu}\left(1 + \ln\left(\frac{y_k}{\mu}\right)\right\}\right]^2}{\mu^2}$$

Mean log deviation

Mean log deviation (MLD) is a relative measure of inequality that considers all population subgroups. Subgroups are weighted according to their population share. MLD measures the extent to which the shares of the population and shares of the health indicator differ across subgroups, weighted by shares of the population. MLD has no unit. It may be more easily interpreted when multiplied by 1000. Greater absolute values indicate higher levels of inequality. MLD is zero if there is no inequality. MLD is calculated as:

$$MLD = \sum_j p_j \left[-\ln\left(\frac{y_j}{\mu}\right)\right] * 1000$$

The variance of MLD is calculated as (2):

$$var(MLD) = \sum_j \frac{p_j{}^2 \sigma_j{}^2}{\mu^2}\left(1 - \frac{1}{y_j/\mu}\right)^2$$

Impact measures

Population attributable fraction

Population attributable fraction (PAF) is a relative measure of inequality that shows the potential improvement in the average of an indicator, in relative terms, that could be achieved if all population subgroups had the same level of the indicator as a reference point (y_{ref}). y_{ref} refers to the most advantaged subgroup for ordered dimensions and the best-performing subgroup for non-ordered dimensions (i.e. the subgroup with the highest value for favourable indicators and is the subgroup with the lowest value for adverse indicators). PAF has no unit. The larger the absolute value of PAF, the higher the level of inequality. PAF is zero if no further improvement can be achieved. PAF is calculated as:

$$PAF = \frac{y_{ref} - \mu}{\mu} * 100$$

The variance of PAF is calculated as (6):

$$var(PAF) = \frac{cN[ad(N-c)+bc^2]}{(a+c)^3(c+d)^3}$$

where *a*, *b*, *c*, *d*, and *N* are numbers of people based on a 2x2 contingency table (Table S3).

Table S3. Contingency table used for the calculation of population attributable fraction variance

		**Indicator**		
		**Achieved**	**Not achieved**	**Total**
**Population subgroup**	**All other subgroups**	a	b	m_1 = a + b
	**Reference subgroup y_{ref}**	c	d	m_2 =c + d
	**Total**	n_1 = a + c	n_2 = b + d	N = a + b + c + d

Population attributable risk

Population attributable risk (PAR) is an absolute measure of inequality that shows the potential improvement in the average of an indicator, in absolute terms, that could be achieved if all population subgroups had the same level of the indicator as a reference point (y_{ref}). y_{ref} refers to the most advantaged subgroup for ordered dimensions and the best-performing subgroup for non-ordered dimensions (i.e. the subgroup with the highest value for favourable indicators and is the subgroup with the lowest value for adverse indicators). PAR has the same unit as the indicator. The larger the absolute value of PAR, the higher the level of inequality. PAR is zero if no further improvement can be achieved. PAR is calculated as:

$$PAR = y_{ref} - \mu$$

The variance of PAR is constructed from the 95% confidence intervals of PAF ($PAF \pm 1.96 PAF_{se}$):

$$var(PAR) = \left[\frac{|\mu(PAF + 1.96PAF_{se}) - (PAF - 1.96PAF_{se})|}{2 * 1.96}\right]^2$$

References

1. Schlotheuber A, Hosseinpoor AR. Summary Measures of Health Inequality: A Review of Existing Measures and Their Application. Int J Environ Res Public Health. 2022 Mar 1;19(6).
2. Ahn J, Harper S, Yu M, Feuer EJ, Liu B, Luta G. Variance Estimation and Confidence Intervals for 11 Commonly Used Health Disparity Measures. JCO Clin Cancer Informatics. 2018 Dec;2(2):1–19.
3. Kakwani NC. Income inequality and poverty: Methods of estimation and policy applications. Popul Dev Rev. 1980;6(4):673.
4. Ahn J, Harper S, Yu M, Feuer EJ, Liu B. Improved Monte Carlo methods for estimating confidence intervals for eleven commonly used health disparity measures. PLoS One. 2019 Jul 1;14(7).
5. Pearcy JN, Keppel KG. A summary measure of health disparity. Public Health Rep. 2002;117(3):273–80.
6. Walter SD. Calculation of Attributable Risks from Epidemiological Data. Int J Epidemiol. 1978 Jun 1;7(2):175–82.
